# Supplementary material for: Exploring the Role of CT-Based Delta-Radiomics in Unresectable Vulvar Cancer
Source: Diagnostics (Basel). 2025 Nov 23;15(23):2972. doi: 10.3390/diagnostics15232972 (PMC12691103; doi:10.3390/diagnostics15232972)
Supplement: Supplementary file 1 [file diagnostics-15-02972-s001.zip › diagnostics-3880663-supplementary.pdf]

Supplementary Tables

Supplementary Table S1. Univariable Cox proportional-hazards regression results for Δ radiomic features versus each of five endpoints; only features with two-sided $p < 0.10$ are shown.

Endpoint	Feature (Abbrev)	HR	95% CI	p-value*
**LC**	GLRLM Run-Length Non-Uniformity Normalized (**RLNU_norm**)	2.6182	[1.05, 6.52]	0.0388
	GLRLM Short-Run Emphasis (**SRE**)	2.6815	[1.02, 7.04]	0.0451
	GLRLM Run Percentage (**RP**)	2.4415	[0.94, 6.33]	0.0663
	GLCM Difference Entropy (**DiffEnt**)	1.7571	[0.96, 3.23]	0.0695
	First-order Entropy	2.1118	[0.91, 4.89]	0.0806
	GLCM Joint Entropy (**JointEnt**)	2.0825	[0.91, 4.75]	0.0810
	GLCM Inverse Difference Moment (**IDM**)	0.4479	[0.17, 1.15]	0.0964
	GLCM Cluster Prominence (**ClusProm**)	1.6013	[0.92, 2.79]	0.0966
**RC**	Shape Maximum 3D Diameter (**Max3D**)	2.3804	[0.97, 5.82]	0.0573
	GLCM Inverse Variance (**InvVar**)	0.5686	[0.30, 1.10]	0.0915
	GLRLM Run Variance (**RunVar**)	1.5355	[0.93, 2.55]	0.0963
	GLRLM Run Percentage (**RP**)	0.5651	[0.29, 1.11]	0.0974
**DMFS**	Shape Surface-Volume Ratio (**SVR**)	2.9078	[1.28, 6.63]	0.0111
	First-order Energy	2.3743	[1.20, 4.68]	0.0126
	GLSZM Size Zone Non-Uniformity (**SZNU**)	2.0364	[1.13, 3.68]	0.0184
	GLDM Gray-Level Non-Uniformity (**GLNU**)	1.9454	[1.06, 3.57]	0.0316
	GLRLM Gray-Level Non-Uniformity (**GLRLM_GLNU**)	1.9562	[1.05, 3.65]	0.0346
	GLRLM Run-Length Non-Uniformity (**RLNU**)	2.0122	[1.05, 3.85]	0.0347
	GLDM Dependence Non-Uniformity (**DNUN**)	1.9431	[1.04, 3.62]	0.0367
	GLSZM Gray-Level Non-Uniformity (**GLSZM_GLNU**)	2.1466	[1.04, 4.45]	0.0401
	GLSZM Zone Variance (**ZSVar**)	1.8474	[1.02, 3.34]	0.0420
	GLSZM Large-Area Emphasis (**LAE**)	1.8453	[1.02, 3.33]	0.0424
	Shape Sphericity	0.4370	[0.19, 1.01]	0.0534
	GLCM Difference Variance (**DiffVar**)	1.7018	[0.97, 2.98]	0.0629
	GLSZM Gray-Level Variance (**GLSZM_GLVar**)	1.6614	[0.95, 2.90]	0.0742
	GLCM Contrast	1.6173	[0.95, 2.75]	0.0759
	GLDM Large Dependence Low Gray-Level Emphasis (**LDLGLE**)	0.0518	[0.00, 1.65]	0.0939
**PFS**	First-order Energy	2.3924	[1.25, 4.58]	0.0086
	GLRLM Run-Length Non-Uniformity (**RLNU**)	2.3074	[1.20, 4.43]	0.0121
	GLDM Dependence Non-Uniformity (**DNUN**)	2.2254	[1.18, 4.20]	0.0136
	GLSZM Large-Area High Gray-Level Emphasis (**LAHGLE**)	1.9793	[1.11, 3.52]	0.0203

	GLRLM Gray-Level Non-Uniformity (**GLRLM_GLNU**)	2.1067	[1.12, 3.96] 0.0205
	Shape Surface-Volume Ratio (**SVR**)	2.0542	[1.10, 3.84] 0.0241
	GLDM Gray-Level Non-Uniformity (**GLNU**)	1.9973	[1.09, 3.67] 0.0260
	GLSZM Zone Variance (**ZSVar**)	1.8950	[1.05, 3.42] 0.0342
	GLSZM Large-Area Emphasis (**LAE**)	1.8949	[1.05, 3.42] 0.0342
	GLSZM Size Zone Non-Uniformity (**SZNU**)	1.7166	[1.03, 2.85] 0.0366
	GLSZM Gray-Level Non-Uniformity (**GLSZM_GLNU**)	2.0021	[1.02, 3.94] 0.0447
	GLSZM Gray-Level Variance (**GLSZM_GLVar**)	1.7531	[1.00, 3.07] 0.0501
	GLSZM Zone Entropy (**ZoneEnt**)	1.8710	[0.99, 3.53] 0.0535
	GLCM Joint Average (**JointAvg**)	1.5006	[0.99, 2.28] 0.0569
	GLCM Sum Average (**SumAvg**)	1.5006	[0.99, 2.28] 0.0569
	First-order Median	0.0167	[0.00, 1.19] 0.0600
	GLDM Dependence Non-Uniformity Normalized (**DNUN_norm**)	1.7032	[0.97, 2.99] 0.0640
	GLCM Inverse Difference Normalized (**IDMN_norm**)	1.8901	[0.94, 3.78] 0.0722
	GLRLM Long-Run High Gray-Level Emphasis (**LRHGLE**)	1.4308	[0.95, 2.16] 0.0900
	Shape Sphericity	0.5613	[0.29, 1.10] 0.0914
	First-order Minimum	1.4114	[0.94, 2.11] 0.0940
	NG TDM Complexity	1.4257	[0.94, 2.16] 0.0952
**OS**	GLSZM Gray-Level Variance (**GLSZM_GLVar**)	2.3442	[1.21, 4.55] 0.0117
	GLSZM Size Zone Non-Uniformity (**SZNU**)	2.1876	[1.19, 4.03] 0.0120
	Shape Surface-Volume Ratio (**SVR**)	3.3452	[1.29, 8.68] 0.0130
	GLCM Difference Variance (**DiffVar**)	2.1517	[1.17, 3.97] 0.0141
	GLCM Contrast	2.0233	[1.14, 3.60] 0.0166
	First-order Root-Mean-Squared (**RMS**)	1.8743	[1.09, 3.23] 0.0236
	First-order Energy	1.7205	[1.04, 2.83] 0.0332
	GLRLM Gray-Level Variance (**RunGLVar**)	1.7209	[1.04, 2.84] 0.0335
	GLSZM Gray-Level Non-Uniformity Normalized (**GLNU_norm**)	0.3154	[0.11, 0.94] 0.0387
	GLCM Cluster Shade	1.8045	[1.01, 3.21] 0.0448
	GLSZM Zone Entropy (**ZoneEnt**)	1.9266	[1.00, 3.70] 0.0490
	First-order Variance	1.6024	[0.98, 2.62] 0.0596
	GLDM Gray-Level Variance	1.5993	[0.98, 2.61] 0.0606
	GLDM Large Dependence Low Gray-Level Emphasis (**LDLGLE**)	0.0219	[0.00, 1.22] 0.0624
	GLCM Sum Squares	1.6046	[0.98, 2.64] 0.0628
	GLCM Cluster Tendency	1.5651	[0.95, 2.58] 0.0799

	GLRLM Long-Run Low Gray-Level Emphasis (**LRLGLE**)	0.0375	[0.00, 1.64]	0.0884
	GLCM Difference Average (**DiffAvg**)	1.7142	[0.91, 3.22]	0.0931
	GLDM Low Gray-Level Emphasis	0.0265	[0.00, 1.88]	0.0951

LC, Local Control; RC, Regional Control; DMFS, Distant Metastasis-Free Survival; PFS, Progression-Free Survival; OS, Overall Survival; GLCM, Gray-Level Co-occurrence Matrix; GLRLM, Gray-Level Run-Length Matrix; GLSZM, Gray-Level Size-Zone Matrix; GLDM, Gray-Level Dependence Matrix; First-order, features derived from the voxel intensity histogram without spatial context.
*Statistical test: Univariable Cox proportional-hazards regression

Supplementary Table S2. Δ radiomic features retained after pairwise Pearson correlation filtering (|r| < 0.90) for each endpoint.

Endpoint	Δ features
**LC (6 Δ features)**	GLCM Inverse Difference Moment (**IDM**)<br>GLRLM Run-Length Non-Uniformity Normalized (**RLNU_norm**)<br>GLRLM Run Percentage (**RP**)<br>First-order Entropy<br>GLCM Difference Entropy (**DiffEnt**)<br>GLCM Cluster Prominence (**ClusProm**)
**RC (3 Δ features)**	GLRLM Run Variance (**RunVar**)<br>Shape Maximum 3D Diameter (**Max3DDiam**)<br>GLCM Inverse Variance (**InvVar**)
**DMFS (8 Δ features)**	Shape Surface-Volume Ratio (**SVR**)<br>GLCM Difference Variance (**DiffVar**)<br>GLDM Gray-Level Non-Uniformity (**GLNU**)<br>GLDM Large Dependence Low Gray-Level Emphasis (**LDLGLE**)<br>GLSZM Size Zone Non-Uniformity (**SZNU**)<br>GLSZM Gray-Level Variance (**GLVar**)<br>Shape Sphericity<br>First-order Energy
**PFS (10 Δ features)**	GLDM Dependence Non-Uniformity Normalized (**DNUN_norm**)<br>Shape Surface-Volume Ratio (**SVR**)<br>GLCM Joint Average (**JointAvg**)<br>GLSZM Size Zone Non-Uniformity (**SZNU**)<br>GLCM Inverse Difference Moment (**IDM**)<br>GLSZM Zone Entropy (**ZoneEnt**)<br>GLSZM Gray-Level Variance (**GLVar**)<br>Shape Sphericity<br>First-order Energy<br>First-order Median
**OS (9 Δ features)**	GLCM Difference Average (**DiffAvg**)<br>Shape Surface-Volume Ratio (**SVR**)<br>GLCM Difference Variance (**DiffVar**)<br>GLDM Large Dependence Low Gray-Level Emphasis (**LDLGLE**)<br>GLSZM Size Zone Non-Uniformity (**SZNU**)<br>GLSZM Gray-Level Non-Uniformity Normalized (**GLNU_norm**)<br>GLSZM Zone Entropy (**ZoneEnt**)

	GLSZM Gray-Level Variance (**GLVar**)<br>First-order Energy

|r| < 0.90, absolute Pearson correlation coefficient (r) below 0.90; LC, Local Control; RC, Regional Control; DMFS, Distant Metastasis-Free Survival; PFS, Progression-Free Survival; OS, Overall Survival; GLCM, Gray-Level Co-occurrence Matrix; GLRLM, Gray-Level Run-Length Matrix; GLSZM, Gray-Level Size-Zone Matrix; GLDM, Gray-Level Dependence Matrix; First-order, features derived from the voxel intensity histogram without spatial context.

Supplementary Table S3. Temporal changes in delta-radiomic features retained in final models

**Feature**	**1st scan median [IQR]**	**2nd scan median [IQR]**	**Δ median [IQR]**	**Direction**	**p-value***
RLNU_norm	0.47 [0.46–0.50]	0.47 [0.44–0.52]	0.00 [–0.07–0.03]	↑	0.478
DiffAvg	0.81 [0.64–1.01]	0.78 [0.66–1.12]	0.00 [–0.05–0.12]	↑	0.351
SVR	0.22 [0.18–0.28]	0.27 [0.21–0.30]	0.09 [0.00–0.29]	↑	0.030
DiffVar	1.80 [1.03–2.72]	1.42 [1.03–4.25]	0.26 [–0.24–0.72]	↑	0.057
LDLGLE	0.14 [0.10–0.30]	0.30 [0.12–0.41]	0.41 [0.00–1.66]	↑	0.100
GLNU_norm	0.10 [0.08–0.12]	0.11 [0.08–0.16]	–0.06 [–0.18–0.37]	↓	0.601
First-order Energy	1.27×10^{8} [2.55×10^{7}–2.30×10^{8}]	4.22×10^{7} [9.20×10^{6}–1.94×10^{8}]	–0.24 [–0.83–1.99]	↓	0.433

RLNU_norm, Run-Length Non-Uniformity Normalized; DiffAvg, Difference Average; SVR, Surface–Volume Ratio; DiffVar, Difference Variance; LDLGLE, Large Dependence Low Gray-Level Emphasis; GLNU_norm, Gray-Level Non-Uniformity Normalized; First-order Energy.
*Statistical test: Paired Wilcoxon signed-rank test.

Supplementary Figures

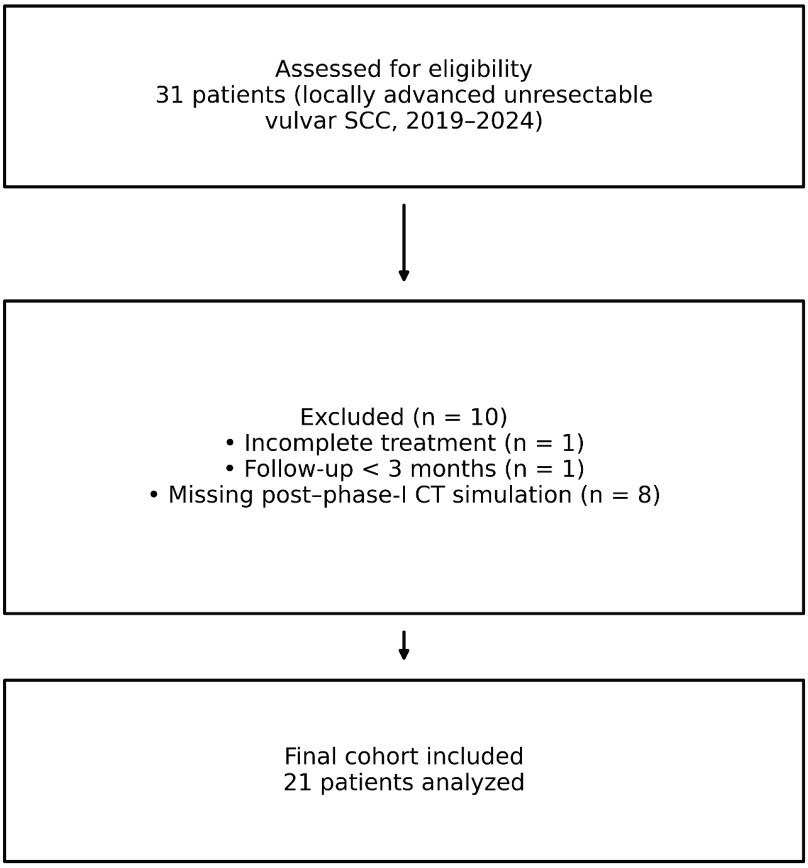

Supplementary Figure S1. Patient inclusion and exclusion flowchart. Between 2019 and 2024, 31 patients with histologically confirmed locally advanced unresectable vulvar squamous cell carcinoma were assessed for eligibility. Ten patients were excluded—one due to incomplete treatment (death during therapy), one with follow-up < 3 months, and eight without a post-phase-I CT simulation scan—leaving 21 patients for the final delta-radiomics analysis.

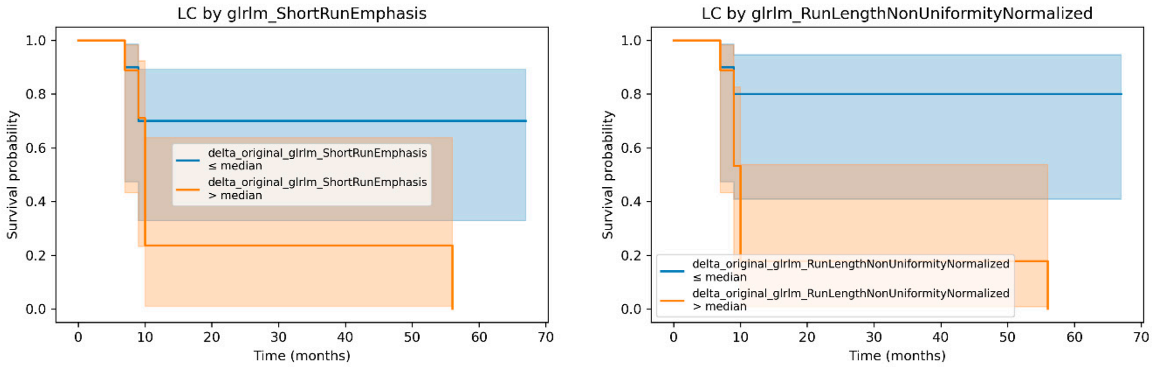

Supplementary Figure S2. On univariable cox regression, two significant Δ features were identified for local control (LC) (see Supplementary Table 1 for hazard ratios and p-values). Kaplan–Meier curves illustrate LC when the cohort is dichotomized at the median value of each feature.

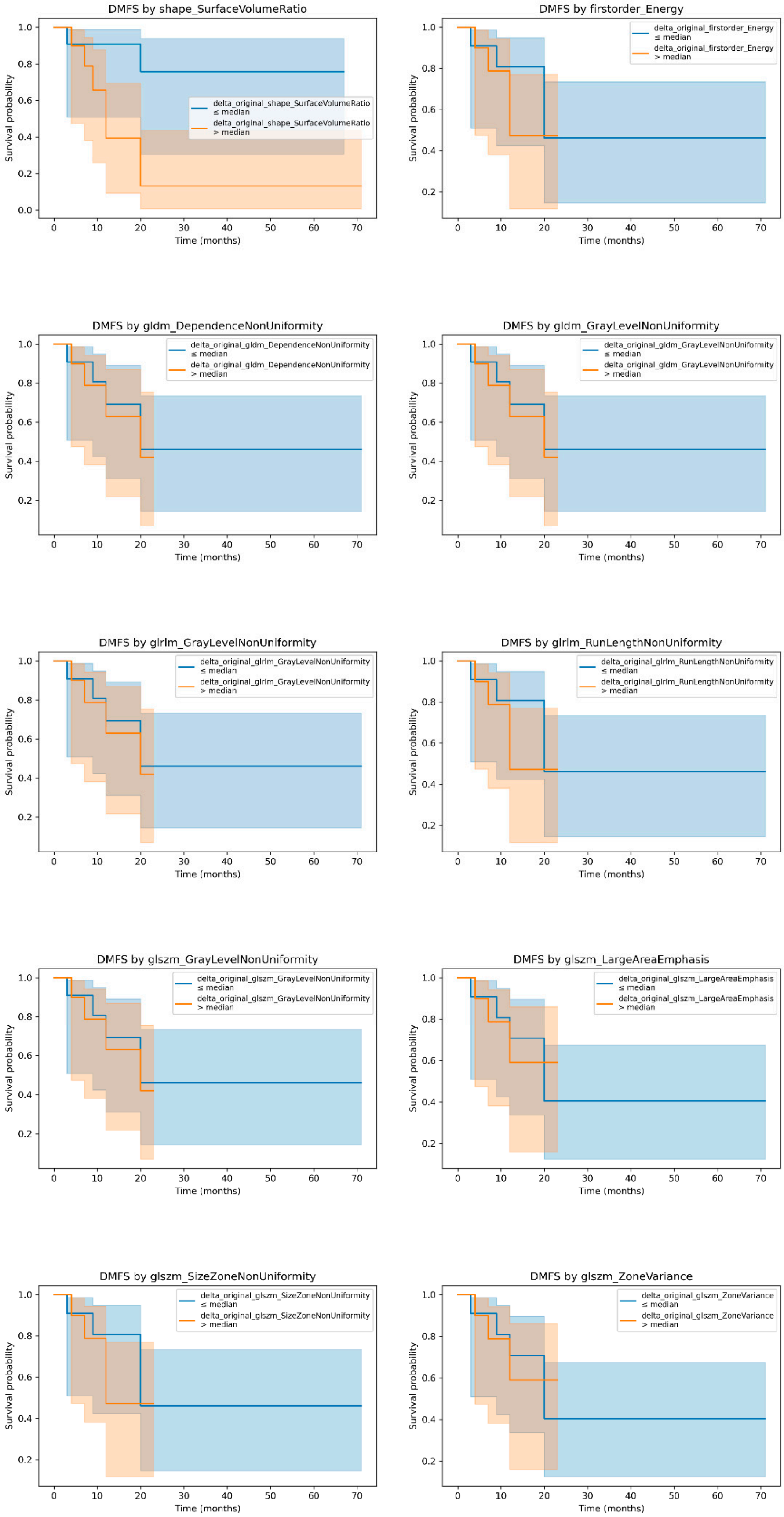

Supplementary Figure S3. On univariable cox regression, several significant Δ features were identified for distant metastasis-free survival (DMFS) (see Supplementary Table 1 for hazard ratios and p-values). Kaplan–Meier curves illustrate DMFS when the cohort is dichotomized at the median value of each feature.

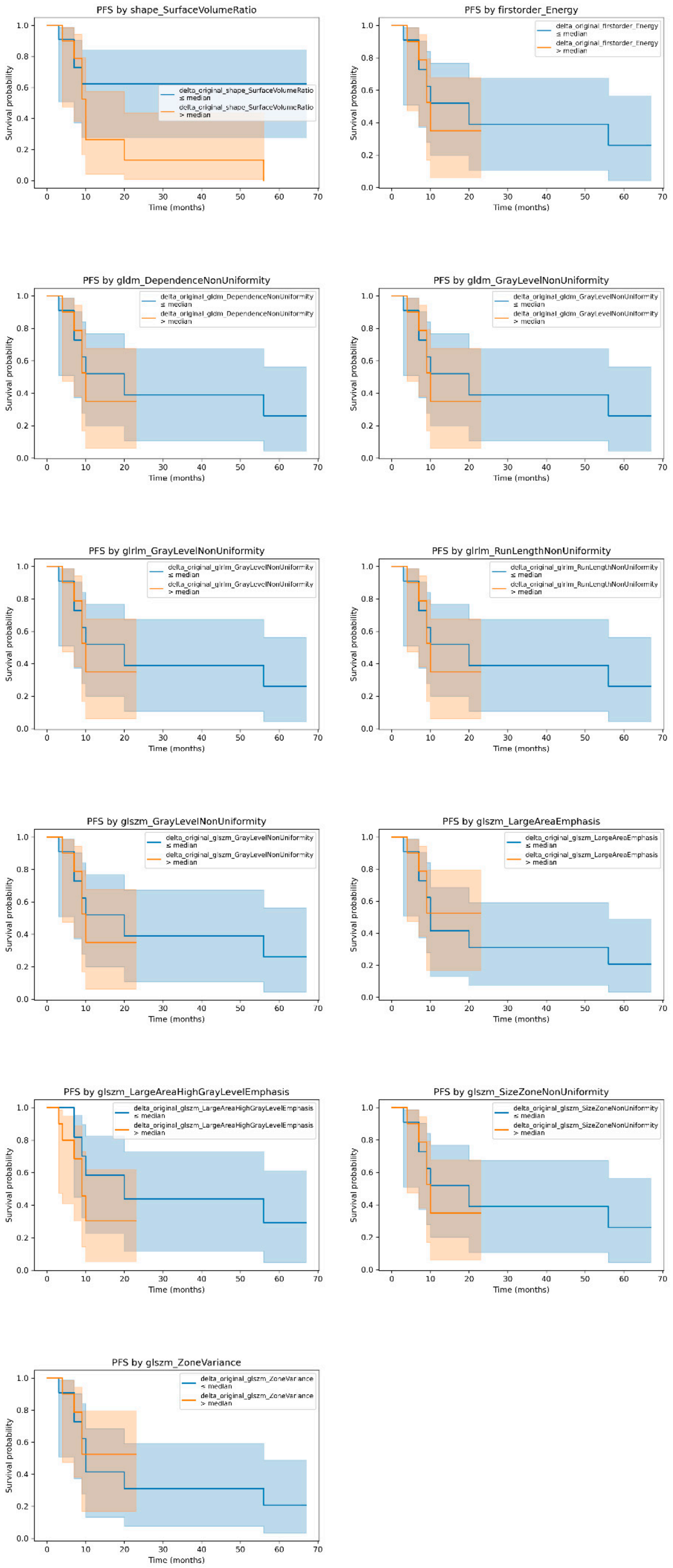

Supplementary Figure S4. On univariable cox regression, several significant Δ features were identified for progression-free survival (PFS) (see Supplementary Table 1 for hazard ratios and p-values). Kaplan–Meier curves illustrate PFS when the cohort is dichotomized at the median value of each feature.

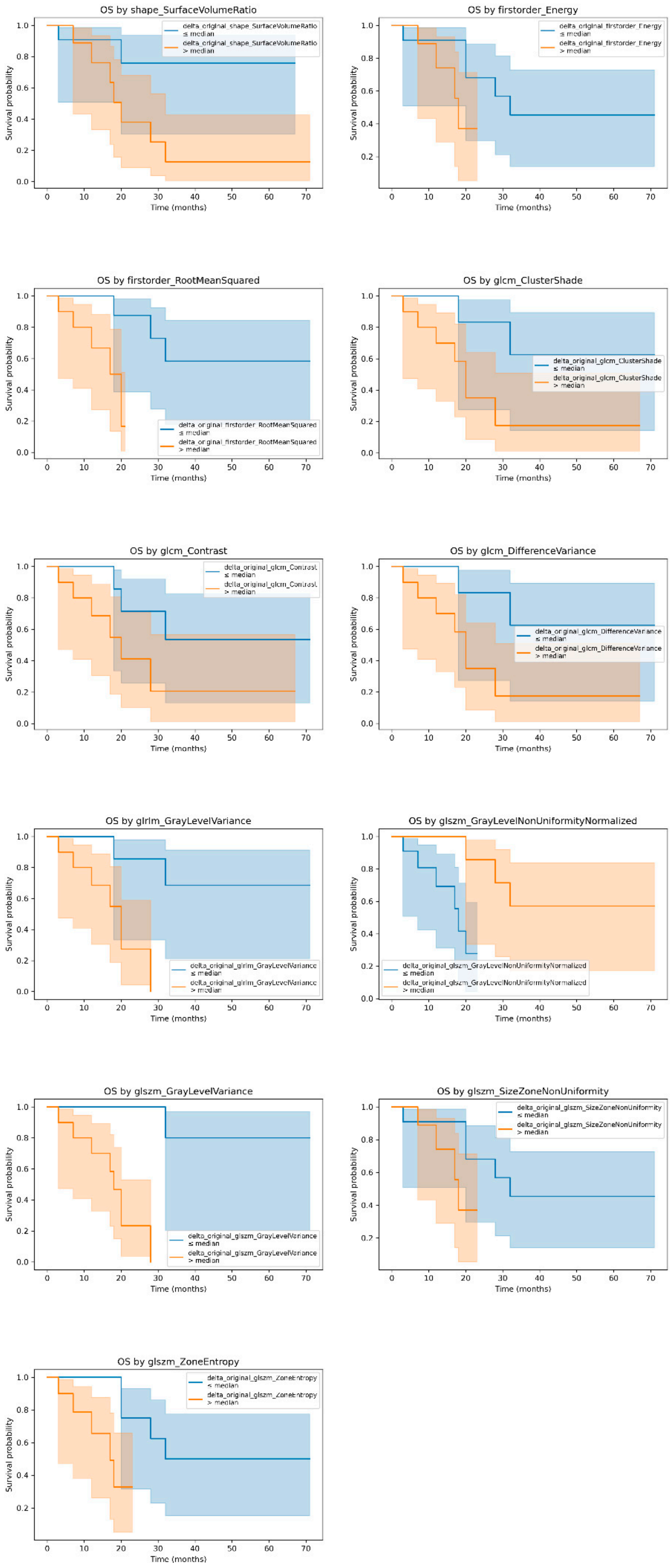

Supplementary Figure S5. On univariable cox regression, several significant Δ features were identified for overall survival (OS) (see Supplementary Table 1 for hazard ratios and p-values). Kaplan–Meier curves illustrate OS when the cohort is dichotomized at the median value of each feature.

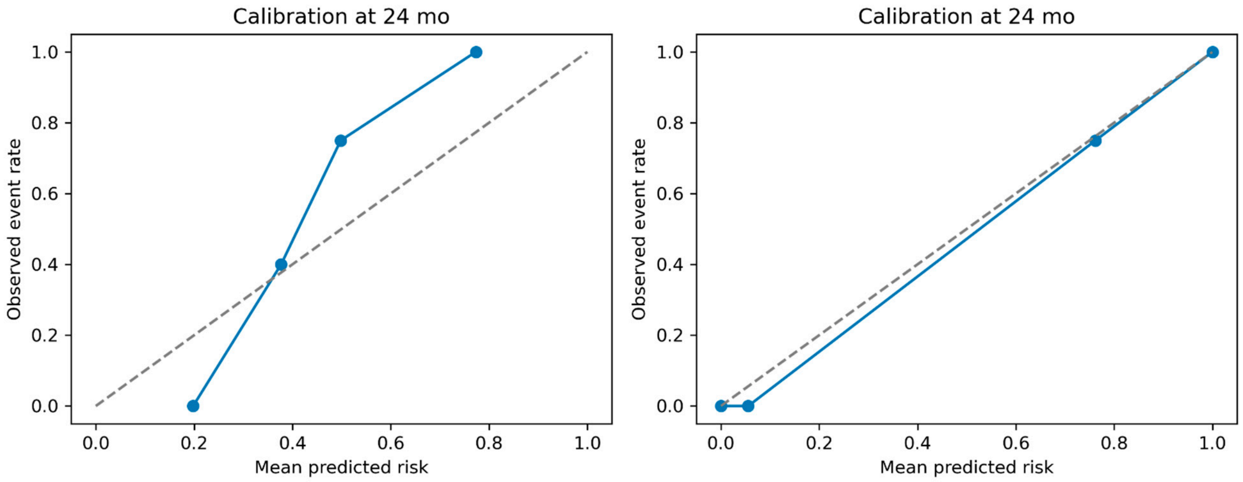

Supplementary Figure S6. Calibration curves for the final multivariable Cox models for local control (LC) and overall survival (OS) at 24 months using Kaplan–Meier–based quartile grouping. For LC model that retained a single feature as predictor, the calibration-in-the-large was −2.889 with a slope of 1.512, indicating underprediction at lower risks and overprediction at higher risks, with observed event rates ranging from 0% to 100% across predicted-risk quartiles. For OS, the calibration-in-the-large was −1.338 with a slope of 0.825, reflecting mild over-dispersion and overall underprediction, with observed event rates also ranging from 0% to 100%. Given the small sample size (n = 21) and low number of events, these results should be interpreted as exploratory.
